# An increase in myeloid cells after severe injury is associated with normal fracture healing: a retrospective study of 62 patients with a femoral fracture

**DOI:** 10.1080/17453674.2018.1501974

**Published:** 2018-08-06

**Authors:** Lillian Hesselink, Okan W Bastian, Marjolein Heeres, Maarten ten Berg, Albert Huisman, Imo E Hoefer, Wouter W van Solinge, Leo Koenderman, Karlijn J P van Wessem, Luke P H Leenen, Falco Hietbrink

**Affiliations:** a Department of Trauma Surgery, University Medical Center Utrecht, Utrecht;; b Department of Clinical Chemistry and Hematology, University Medical Center Utrecht, Utrecht;; c Laboratory for Translational Immunology and Department of Respiratory Medicine, University Medical Center Utrecht Wilhelmina Children’s Hospital, Utrecht, The Netherlands

## Abstract

Background and purpose—Nonunion is common in femoral fractures. Previous studies suggested that the systemic immune response after trauma can interfere with fracture healing. Therefore, we investigated whether there is a relation between peripheral blood cell counts and healing of femur fractures.

Patients and methods—62 multi-trauma patients with a femoral fracture presenting at the University Medical Centre Utrecht between 2007 and 2013 were retrospectively included. Peripheral blood cell counts from hematological analyzers were recorded from the 1st through the 14th day of the hospital stay. Generalized estimating equations were used to compare outcome groups.

Results—12 of the 62 patients developed nonunion of the femoral fracture. The peripheral blood-count curves of total leukocytes, neutrophils, monocytes, lymphocytes, and platelets were all statistically significantly lower in patients with nonunion, coinciding with significantly higher CRP levels during the first 2 weeks after trauma in these patients.

Interpretation—Patients who developed femoral nonunion after major trauma demonstrated lower numbers of myeloid cells in the peripheral blood than patients with normal fracture healing. This absent rise of myeloid cells seems to be related to a more severe post-traumatic immune response.

Nonunion has been reported in one-tenth of patients with femoral fractures. This risk further increases in cases of multiple fractures and open fractures, as frequently seen in multi-trauma (Zura et al. 2016).

Local factors, such as severe soft tissue injury and reduced weight-bearing on the affected extremity, can impair bone healing (Karladani et al. 2001, Taitsman et al. 2009, Zura et al. 2016). In addition, an increasing body of evidence suggests that the systemic immune response can also influence bone healing (Bastian et al. 2011). For instance, blunt chest injury in an experimental setting or intraperitoneal injection of lipopolysaccharides, which are both models for systemic inflammation, impaired fracture healing in animal models (Reikerås et al. 2005, Claes et al. 2011, Recknagel et al. 2013). However, the exact mechanism underlying the fracture healing impairment after systemic inflammation remains unknown.

Secondary bone healing consists of at least 4 different stages: the inflammatory phase, soft callus formation, hard callus formation, and tissue remodeling. During the inflammatory phase, neutrophils and macrophages are recruited to the fracture hematoma within days up to a week after injury (Li et al. 2016, Loi et al. 2016). The inflammatory phase normally ends within a week, after which the formation of callus starts (Marsell and Einhorn 2011, Loi et al. 2016). Disruption of the inflammatory process, for example by sustained inflammation, may interfere with the consecutive stages of bone healing and, thereby, increase the risk of nonunion (Loi et al. 2016).

It is unclear if a correlation exists between the cellular systemic immune response after trauma and femoral fracture healing. Hence, we investigated whether peripheral blood cell counts differ between multi-trauma patients with normal and impaired fracture healing of the femur.  

## Patients and methods

### Study design and setting

Patients were selected from the trauma registry database of the University Medical Center (UMC) Utrecht which collected data of all patients who were admitted to the trauma department. Patients admitted between January 1, 2007 and the December 31, 2013, were included. Hemoglobin concentrations and total number of leukocytes, neutrophils, monocytes, platelets, erythro­cytes and lymphocytes were compared during the first 2 weeks after injury between multi-trauma patients with nonunion and multi-trauma patients with normal healing of the femoral fracture. In addition, the acute phase protein C-reactive protein (CRP) and reticulocyte counts were obtained. Outcome data concerning healing of the femoral fracture were obtained from the electronic medical record system 1 year after trauma.

### Participants

Multi-trauma patients ≥16 years of age with a femoral shaft or distal femoral fracture presenting in the emergency department (ED) of the UMC Utrecht were included. Exclusion criteria were: (1) transfer to another hospital and (2) non-weight bearing of the affected extremity, for instance due to paresis, amputation, or severe head injury. Also, patients were excluded if the healing outcome could not be determined due to death or loss to follow-up. Data concerning patient characteristics, trauma mechanism, injuries, and treatment were obtained from the trauma registry database and supplemented with information from the electronic medical record system.

**Figure 1.**
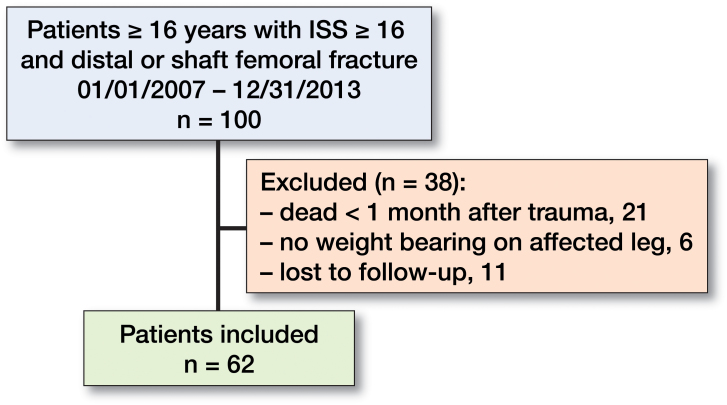
Flowchart of patients who met inclusion/exclusion criteria for the study population. ISS = Injury Severity Score.

100 multi-trauma patients with a distal or shaft femoral fracture were enrolled in the study (Figure 1). Multi-trauma was defined as an Injury Severity Score (ISS) ≥ 16 (Baker et al. 1974). 11 patients were lost to follow-up, of which 8 were transferred to another hospital, 2 did not visit the outpatient clinic, and in one case the information in the patient record system was insufficient to determine healing outcome. 21 patients died. Causes of death were severe traumatic injuries (n = 18), inflammatory complications (n = 2), and unknown (n = 1). 6 patients were excluded because of the inability to bear weight on the affected leg due to severe ipsilateral injuries, an amputation, severe head trauma, or spinal cord injury. Of the remaining 62 patients, union of the femoral fracture was seen in 50 patients (50/62) and nonunion was seen in 12 patients (Table).

**Figure 2.**
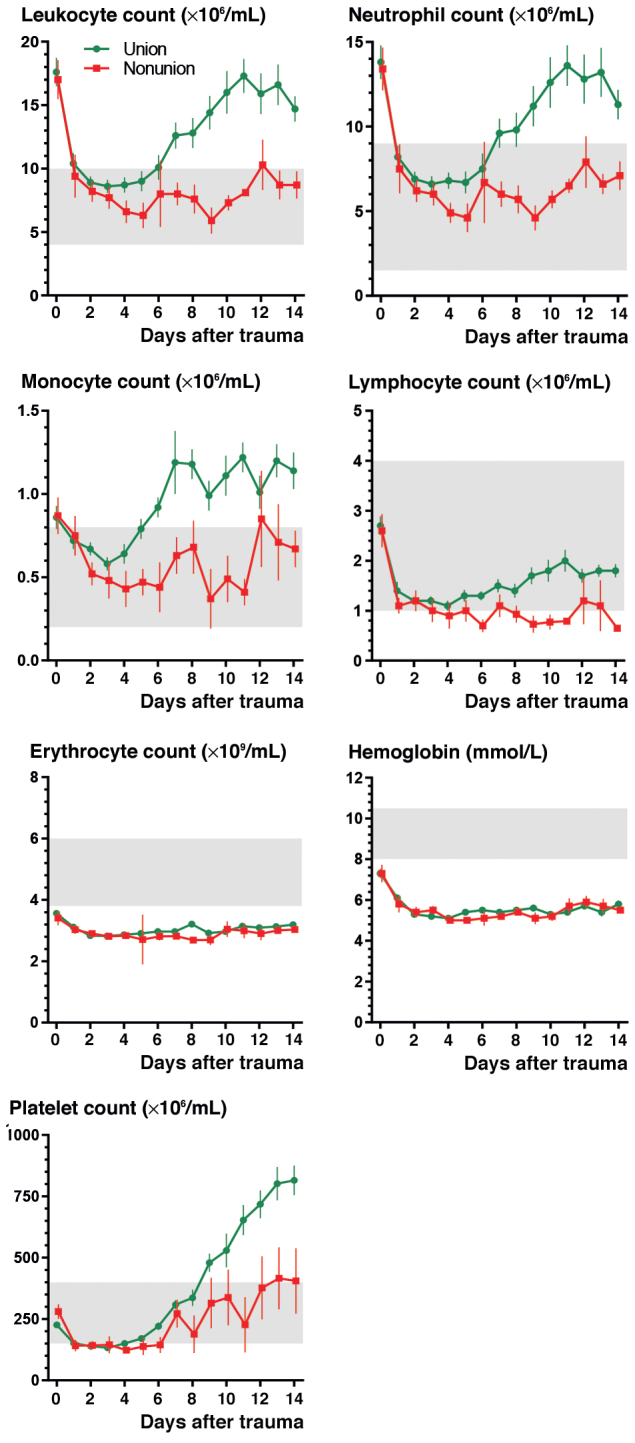
Leukocyte count (A), neutrophil count (B), monocyte count (C), lymphocyte count (D), erythrocyte count (E), hemoglobin (F), and platelet count (G) during the first 2 weeks after major trauma. Patients with union are depicted in green and patients with nonunion in red. Data are presented as mean with standard error of the mean. Grey bars represent reference values.

**Table:** Table: Baseline characteristics. Data are shown as number (percentage), median [range] or mean [standard deviation]

Factor	Total		Union		Nonunion		
	(n = 62)		(n = 50)		(n = 12)		p-value
Sex, male	46	(74)	38	(76)	8	(67)	0.5
Age	32	[16–85]	30	[16–85]	40	[11.7]	0.2
Injury Severity Score	25	[17–48]	26	[17–48]	24	[4.9]	0.3
New Injury Severity Score	27	[17–50]	27	[17–50]	28	[6.2]	0.6
Femur fracture localization							
Shaft	43	(69)	36	(72)	7	(58)	0.5
Distal	19	(31)	14	(28)	5	(42)	0.5
Type of femur fractures (AO):							
Simple extra-articular	27	(44)	29	(60)	6	(50)	0.8
Complex extra-articular	21	(34)	10	(20)	3	(25)	0.7
Intra-articular	14	(23)	11	(22)	3	(25)	1.0
Soft tissue injury (Gustilo)							
0: closed fracture	35	(57)	29	(58)	6	(50)	0.5
1: wound <1cm	8	(13)	5	(10)	3	(25)	0.2
2: wound >1 cm	6	(10)	5	(10)	1	(8)	1.0
3a: adequate soft tissue cover	4	(7)	3	(6)	1	(8)	1
3b: inadequate soft tissue cover	2	(3)	2	(4)	0		1
3c: associated arterial injury	0		0		0		
Unknown	7	(11)	6	(12)	1	(8)	
Type of fixation							
External fixation + IMN	6	(10)	5	(10)	1	(8)	1
External fixation + plates	9	(15)	7	(14)	2	(17)	1
External fixation + screws	1	(2)	1	(2)	0		1
IMN	35	(57)	30	(60)	5	(42)	0.4
Plates	10	(16)	6	(12)	4	(33)	0.1
Screws	1	(2)	1	(2)	0		1
Number of surgical procedures	3	[1–18]	3	[1–13]	3	[1–18]	0.4
Inflammatory complications							
Urinary tract infection	6	(10)	5	(10)	1	(8)	1
Surgical site infection	3	(5)	2	(4)	1	(8)	0.5
Pneumonia	10	(16)	9	(18)	1	(8)	0.7
MODS	3	(5)	2	(4)	1	(8)	0.5
Nonunion	13	(21)					
Atrophic	11	(18)			11	(92)	
Hypertrophic	1	(2)			1	(8)	
Infected nonunion	2	(3)			2	(17)	
ICU stay (days)	2	[0–68]	2	[0–46]	0	[0–68]	0.2
Hospital stay (days)	20	[4–154]	20	[4–95]	16	[4–154]	0.3

Baseline variables of patients with nonunion are compared with baseline variables of patients with union with the use of Fisher’s exact test or Mann-Whitney U test as indicated. IMN = intramedullary nailing, ICU = intensive care unit, MODS = multiple organ dysfunction syndrome.

### Procedures

Hematological parameters were obtained from the Utrecht Patient Oriented Database (UPOD). Data were collected from the day patients arriving in the Emergency Department (ED) through the 14th day of their hospital stay. The technical details of the UPOD are described elsewhere (ten Berg et al. 2007). In short, this database is an infrastructure of relational databases that allows (semi-)automated transfer, processing and storage of data, including administrative information, medical and surgical procedures, medication orders, and laboratory test results for all clinically admitted patients and patients attending the outpatient clinic of the UMC Utrecht since 2004. The process and storage of data are in accordance with privacy and ethics regulations. UPOD data acquisition and data management are in line with current Dutch regulations concerning privacy and ethics and are approved by the institution’s medical ethics committee. Because no extra material, such as blood samples, was taken from patients, there was no requirement to obtain informed consent from individual patients. The data were analyzed anonymously. Routine hematological analysis was performed using the Cell-Dyn Sapphire hematology analyzer (Abbott Diagnostics, Santa Clara, CA, USA) (ten Berg et al. 2007). The reliability and validity of the laboratory results were monitored through routine quality control.

### Variables and outcome measures

The study outcome was femoral fracture healing. Union was defined as pain-free mobilization (clinical union) or bridging of 3 of the 4 cortices (radiological union) within 12 months after injury. Nonunion was defined as lack of radiological and clinical union within 12 months after trauma or a fracture which required a re-intervention to achieve union. Peripheral blood cell counts of multi-trauma patients with union were compared with peripheral blood cell counts of multi-trauma patients with nonunion. Soft tissue injury was scored according to the Gustilo classification (Gustilo et al. 1984).

### Statistics

Data were analyzed with IBM SPSS version 23 (IBM Corp, Armonk NY, USA). Descriptive statistics are presented as median (range) for non-normally distributed variables and mean (SD) for normally distributed variables. Comparison of baseline variables between outcome groups was performed with Fisher’s exact test for categorical variables or a Mann–Whitney U test for the continuous data that were not normally distributed. Statistical significance was defined as a p-value <0.05. Since the design of the study is longitudinal with repeated measurements, we chose linear generalized estimating equations (GEE) to compare the development over time of hematological parameters between outcome groups. This linear analysis was performed to analyze whether the course of hematological parameters differed between the outcome groups during the first 2 weeks after trauma. The GEE was used to account for within-subject correlation between repeated measurements. Based on spaghetti plots we chose the autoregressive working correlation structure for platelets and the exchangeable working correlation structure for the other hematological parameters.

### Ethics, funding, and potential conflicts of interest

A waiver was provided by the institutional medical ethics committee for this study. In addition, in line with the academic hospital policy, an opt-out procedure is in place for use of patient data for research purposes. The process and storage of data are in accordance with privacy and ethics regulations. Financial support was not received. No conflicts of interests were declared.

## Results

### Demographics

There were no statistically significant differences in sex, age, ISS, new injury severity score (NISS), fracture localization, type of fracture, soft tissue injury, type of fixation, number of surgical procedures, inflammatory complications, length of stay in intensive care unit (ICU), and total hospital stay between patients with normal and impaired fracture healing of the femur.  

### Peripheral blood cell counts (Figure 2)

Neutrophil and leukocyte counts were similarly elevated in both outcome groups upon arrival in ED and decreased to normal values within 1 day. In patients with union, mean leukocyte, neutrophil, monocyte, and platelet counts rose above reference values in the second week after trauma. In contrast, leukocyte, neutrophil, monocyte, and platelet counts of patients with nonunion remained within reference values. Lymphocyte counts remained within reference values in patients with union and decreased to just below reference values in patients with nonunion. When compared with the union group, in the nonunion group there was an average change in leukocytes of –0.33/day (p = 0.03), neutrophils of –0.39/day (p = 0.03), monocytes of –0.03/day (p = 0.03), platelets of –21/day (p = 0.001) and lymphocytes of –0.04/day (p = 0.02). Hemoglobin and erythrocytes decreased after trauma and remained below reference values for both outcome groups. 

### Reticulocyte count and CRP level (Figure 3)

**Figure 3.**
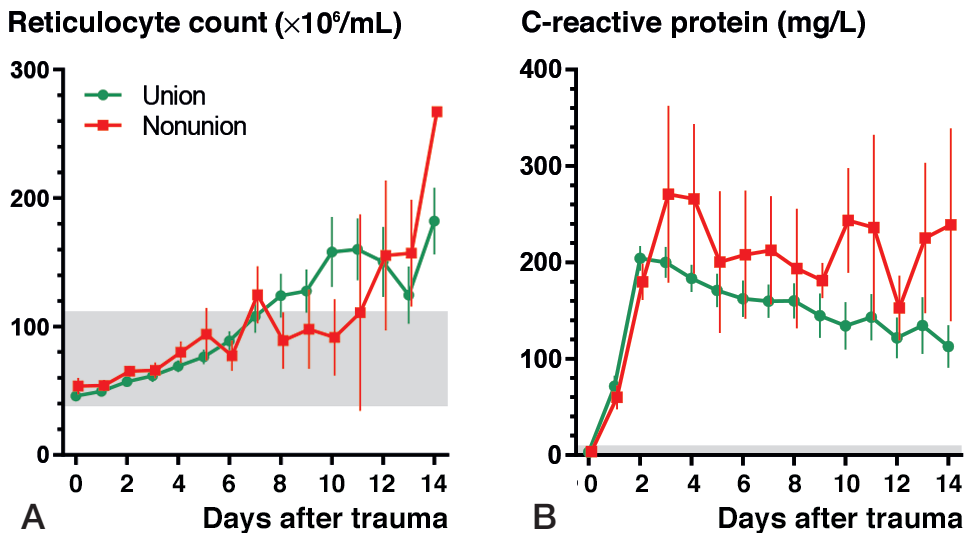
Reticulocyte count (A) and C-reactive protein (B) during the first 2 weeks after major trauma. Patients with union are depicted in green and patients with nonunion in red. Data are presented as mean with standard error of the mean. Grey bars represent reference values.

Figure 3 show reticulocyte counts and CRP levels during the first 2 weeks after trauma for patients with union and nonunion of the femoral fracture. In both outcome groups, CRP levels rose to 200–300 within 3 days, and gradually decreased thereafter. After day 2, higher CRP levels were observed in patients who later developed nonunion than in patients with union. The average change in CRP levels in patients with nonunion was 7.4/day (p = 0.01) compared with CRP levels of patients with union. No statistically significant differences were observed between outcome groups in the number of infections and the number of severe infections leading to multiple organ dysfunction syndrome. Reticulocyte count rose in both outcome groups after trauma, and was similar in patients with union and patients with nonunion. 

## Discussion

Leukocytes, neutrophils, monocytes, and platelets were above reference values in patients with normal fracture healing during the second week after severe injury. Patients with nonunion did not exhibit such an increase in myeloid blood cells and exhibited a statistically significant, but minor, decrease in lymphocytes. Although CRP levels were elevated in both outcome groups, there was a small but statistically significant increase in CRP in the nonunion group compared with the union group.

An increase of myeloid cells after trauma, as seen in patients with union, has been described before (Manz and Boettcher 2014, Bastian et al. 2016b, Loftus et al. 2017). Moreover, a previous study found similar trends in peripheral blood cell counts in patients with and without union of their tibia fracture (Bastian et al. 2016b). This study additionally investigated CRP and reticulocyte count. CRP provided information on inflammation and reticulocyte count reflected the production of immature red blood cells from the bone marrow, and can thus be used as an indicator of bone marrow function (Livingston et al. 2003, Piva et al. 2015). There are different hypotheses that can explain the lack of leukocytosis and thrombocytosis in the nonunion group. First, persistent inflammation might suppress the bone marrow response (Livingston et al. 2003). However, the increase found in reticulocytes, which did not differ between outcome groups, suggests an adequate bone marrow response and makes this hypothesis less likely. Second, the lack of leukocytosis and thrombocytosis might be caused by persistent extravasation of myeloid cells to the tissues, a process associated with inflammatory conditions (Hietbrink et al. 2006, Johansson 2014).

Both hypotheses regarding the lack of leukocytosis and thrombocytosis in nonunion patients without bone marrow suppression are based on sustained inflammation. This is supported by the finding that nonunion patients had slightly higher CRP levels, while there was no statistically significant difference in clinically evident infections between outcome groups. Previous studies have demonstrated that a local controlled inflammatory reaction is key to successful bone healing (Schell et al. 2017) and that sustained systemic inflammation after trauma can impair this process (Recknagel et al. 2011, Claes et al. 2011). The influx of leukocytes in the fracture hematoma is an essential step during the inflammatory phase in the first week after trauma (Marsell and Einhorn 2011, Bastian et al. 2016a). However, termination of this phase to prevent persistent inflammation seems to be at least as important (Schmidt-Bleek et al. 2012, Loi et al. 2016). Decreased numbers of myeloid cells in the blood of nonunion patients during the second week after trauma supports the hypothesis of enhanced extravasation of these cells after the inflammatory phase and thus persistent local inflammation. This is in line with previous studies showing a relation between impaired fracture healing and increased numbers of pro-inflammatory leukocytes in the fracture hematoma (Schmidt-Bleek et al. 2012) and decreased numbers of leukocytes in the peripheral blood during the second week after trauma (Bastian et al. 2016b). Furthermore, previous studies showed that both the reduction of neutrophils in the fracture hematoma, and the inhibition of extravasation by blocking the anaphylatoxin C5a, improved fracture healing in rats (Grogaard et al. 1990, Chung et al. 2006, Recknagel et al. 2012). Taken together, it is tempting to speculate that increased extravasation of myeloid cells can disturb fracture healing and that this is reflected by decreased numbers of myeloid cells in the peripheral blood.

It is not surprising that conditions which further enhance the post-traumatic immune response, such as open fractures and multiple injuries, are additional risk factors for nonunion. However, we did not find statistically significant differences in soft tissue injury and injury severity between the two outcome groups. Other factors that can influence peripheral blood cell counts, such as infectious complications and the number of surgical procedures, were also not significantly different.

An important limitation of this study is that blood values were retrospectively obtained and were therefore not available for each patient at each time point. It is possible that blood was more frequently drawn from patients who were more severely injured or from patients who developed complications during hospital stay. Yet, we did not find a relation between ISS or complications and healing outcome, precluding a substantial bias.

In summary, multi-trauma patients who developed femoral nonunion after major trauma demonstrated lower numbers of myeloid cells in the peripheral blood than patients with normal fracture healing. Patients with union demonstrated leukocyte numbers above reference values in the second week after trauma, reflecting a normal physiological response. These findings support the hypothesis that persistent systemic inflammation after major injury can affect physiological processes necessary for bone healing.

FH, LPHL, KJPW, LK, MH, and LH developed the original study design. AH, MB, IEH, WWS, and LH contributed to the data acquisition. OWB, FH, and LH contributed to the analysis and drafted the paper.

*Acta* thanks Anita Ignatius and other anonymous reviewers for help with peer review of this study.

## References

[CIT0001] Baker S P, O’Neill B, Haddon W, Long W B The injury severity score: a method for describing patients with multiple injuries and evaluating emergency care. J Trauma 1974; 14(3): 187–96. 4814394

[CIT0002] Bastian O, Pillay J, Alblas J, Leenen L, Koenderman L, Blokhuis T Systemic inflammation and fracture healing. J Leukoc Biol 2011; 89(5): 669–73. 21208896 10.1189/jlb.0810446

[CIT0003] Bastian O W, Koenderman L, Alblas J, Leenen L P H, Blokhuis T J Neutrophils contribute to fracture healing by synthesizing fibronectin + extracellular matrix rapidly after injury. Clin Immunol2016a; 164: 78–84. 26854617 10.1016/j.clim.2016.02.001

[CIT0004] Bastian O W, Kuijer A, Koenderman L, Stellato R K, van Solinge W W, Leenen L P H, et al. Impaired bone healing in multitrauma patients is associated with altered leukocyte kinetics after major trauma. J Inflamm Res2016b; 9: 69–78. 27274302 10.2147/JIR.S101064 PMC4876940

[CIT0005] Chung R, Cool J C, Scherer M A, Foster B K, Xian C J Roles of neutrophil-mediated inflammatory response in the bony repair of injured growth plate cartilage in young rats. J Leukoc Biol 2006; 80(6): 1272–80. 16959896 10.1189/jlb.0606365

[CIT0006] Claes L, Ignatius A, Lechner R, Gebhard F, Kraus M, Baumgärtel S, et al. The effect of both a thoracic trauma and a soft-tissue trauma on fracture healing in a rat model. Acta Orthop 2011; 82(2): 223–7. 21463222 10.3109/17453674.2011.570677 PMC3235295

[CIT0007] Grogaard B, Gerdin B, Reikerfis O The polymorphonuclear leukocyte: has it a role in fracture healing? Arch Orthop Trauma Surg 1990; 109(5): 268–71. 2271360 10.1007/BF00419942

[CIT0008] Gustilo R B, Mendoza R M, Williams D N Problems in the management of type III (severe) open fractures: a new classification of type III open fractures. J Trauma 1984; 24(8): 742–6. 6471139 10.1097/00005373-198408000-00009

[CIT0009] Hietbrink F, Koenderman L, Rijkers G, Leenen L Trauma: the role of the innate immune system. World J Emerg Surg 2006; 1: 15. 16759367 10.1186/1749-7922-1-15 PMC1481567

[CIT0010] Johansson M W Activation states of blood eosinophils in asthma. Clin Exp Allergy 2014; 44(4): 482–98. 24552191 10.1111/cea.12292 PMC4057046

[CIT0011] Karladani A H, Granhed H, Kärrholm J, Styf J The influence of fracture etiology and type on fracture healing: a review of 104 consecutive tibial shaft fractures. Arch Orthop Trauma Surg 2001; 121(6): 325–8. 11482464 10.1007/s004020000252

[CIT0012] Li H, Liu J, Yao J, Zhong J, Guo L, Sun T Fracture initiates systemic inflammatory response syndrome through recruiting polymorphonuclear leucocytes. Immunol Res 2016; 64(4): 1053–9. 27167071 10.1007/s12026-016-8801-2

[CIT0013] Livingston D H, Anjaria D, Wu J, Hauser C J, Chang V, Deitch E A, et al. Bone marrow failure following severe injury in humans. Ann Surg 2003; 238(5): 748–53. 14578739 10.1097/01.sla.0000094441.38807.09 PMC1356155

[CIT0014] Loftus T J, Mohr A M, Moldawer L L Dysregulated myelopoiesis and hematopoietic function following acute physiologic insult. Curr Opin Hematol 2017; 25(1): 37–43. 10.1097/MOH.0000000000000395 PMC5733709 29035909

[CIT0015] Loi F, Córdova L A, Pajarinen J, Lin T hua, Yao Z, Goodman S B Inflammation, fracture and bone repair. Bone 2016; 86: 119–30. 26946132 10.1016/j.bone.2016.02.020 PMC4833637

[CIT0016] Manz M G, Boettcher S Emergency granulopoiesis. Nat Rev Immunol 2014; 14(5): 302–14. 24751955 10.1038/nri3660

[CIT0017] Marsell R, Einhorn T The biology of fracture healing. Injury 2011; 42(6): 551–5. 21489527 10.1016/j.injury.2011.03.031 PMC3105171

[CIT0018] Piva E, Brugnara C, Spolaore F, Plebani M Clinical utility of reticulocyte parameters. Clin Lab Med 2015; 35(1): 133–63. 25676377 10.1016/j.cll.2014.10.004

[CIT0019] Recknagel S, Bindl R, Kurz J, Wehner T, Ehrnthaller C, Knöferl M W, et al. Experimental blunt chest trauma impairs fracture healing in rats. J Orthop Res 2011; 29(5): 734–9. 21437953 10.1002/jor.21299

[CIT0020] Recknagel S, Bindl R, Kurz J, Wehner T, Schoengraf P, Ehrnthaller C, et al. C5aR-antagonist significantly reduces the deleterious effect of a blunt chest trauma on fracture healing. J Orthop Res 2012; 30(4): 581–6. 21922535 10.1002/jor.21561 PMC3244519

[CIT0021] Recknagel S, Bindl R, Brochhausen C, Göckelmann M, Wehner T, Schoengraf P, et al. Systemic inflammation induced by a thoracic trauma alters the cellular composition of the early fracture callus. J Trauma Acute Care Surg 2013; 74(2): 531–7. 23354247 10.1097/TA.0b013e318278956d

[CIT0022] Reikerås O, Shegarfi H, Wang JE, Utvåg S E Lipopolysaccharide impairs fracture healing: an experimental study in rats. Acta Orthop 2005; 76(6): 749–53. 16470425 10.1080/17453670510045327

[CIT0023] Schell H, Duda G N, Peters A, Tsitsilonis S, Johnson K A, Schmidt-Bleek K The haematoma and its role in bone healing. J Exp Orthop 2017; 4(1): 5. 28176273 10.1186/s40634-017-0079-3 PMC5296258

[CIT0024] Schmidt-Bleek K, Schell H, Schulz N, Hoff P, Perka C, Buttgereit F, et al. Inflammatory phase of bone healing initiates the regenerative healing cascade. Cell Tissue Res 2012; 347(3): 567–73. 21789579 10.1007/s00441-011-1205-7

[CIT0025] Taitsman L A, Lynch J R, Agel J, Barei D P Risk factors for femoral nonunion after femoral shaft fracture. J Trauma 2009; 67(6): 1389–92. 19704386 10.1097/TA.0b013e318182afd0

[CIT0026] ten Berg M J, Huisman A, Van den Bemt P M L A, Schobben A F A M, Egberts A C G, van Solinge W W Linking laboratory and medication data: new opportunities for pharmacoepidemiological research. Clin Chem Lab Med 2007; 45(1): 13–19. 17243908 10.1515/CCLM.2007.009

[CIT0027] Zura R, Xiong Z, Einhorn T, Watson J T, Ostrum R F, Prayson M J, et al. Epidemiology of fracture nonunion in 18 human bones. JAMA Surg 2016; 151(11): e162775. 27603155 10.1001/jamasurg.2016.2775

